# Expression of ACE2 and a viral virulence-regulating factor CCN family member 1 in human iPSC-derived neural cells: implications for COVID-19-related CNS disorders

**DOI:** 10.1186/s41232-020-00143-6

**Published:** 2020-09-11

**Authors:** Yoshitaka Kase, Hideyuki Okano

**Affiliations:** 1 Department of Physiology, Keio University School of Medicine, 35 Shinanomachi, Shinjuku-ku, Tokyo, 160-8582 Japan; 2 Department of Geriatric Medicine, Graduate School of Medicine, The University of Tokyo, Bunkyo-ku, Tokyo, 113-8655 Japan

**Keywords:** ACE2, CCN1 (Cyr61), CNS disorder, COVID-19, hiPSC-NS/PCs, SARS-CoV-2

## Abstract

It has been reported that coronavirus disease 2019 (COVID-19) causes not only pneumonia but also systemic inflammations including central nervous system (CNS) disorders. However, little is known about the mechanism that triggers the COVID-19-associated CNS disorders, due to the lack of appropriate experimental systems. Our present study showed that angiotensin-converting enzyme-2 (ACE2), a cellular receptor for SARS-CoV-2, is expressed in human induced pluripotent stem cell (iPSC)-derived neural stem/progenitor cells (hiPSC-NS/PCs) and young neurons. Furthermore, together with database analysis, we found that a viral virulent factor CCN family member 1 (CCN1), which is known to be induced by SARS-CoV-2 infection, is expressed in these cells at basal levels. Considering the role of CCN1 which is known to be involved in viral toxicity and inflammation, hiPSC-NS/PCs could provide an excellent model for COVID-19-associated CNS disorders from the aspect of SARS-CoV-2 infection-ACE2-CCN1 axis. In addition, we identified compounds that reduce CCN1 expression. Collectively, our study using hiPSC-NS/PCs may aid in the development of a therapeutic target for COVID-19-related CNS disorders.

## Background

Severe acute respiratory syndrome coronavirus 2 (SARS-CoV-2) is spreading worldwide at the largest scale and fastest speed [1]. As of June 2020, the cumulative number of infected people was more than 9.6 million, with more than 490,000 deaths due to coronavirus disease 2019 (COVID-19) [2]. SARS-CoV-2-infected cells which express angiotensin-converting enzyme-2 (ACE2) and TMPRSS2 [3, 4]. ACE2 is expressed in epithelial cells of the oral mucosa [5], respiratory tract including the lungs [4], and digestive tract [6]. The virus specifically infects these cells with strong toxicity, which causes various symptoms, such as pneumonia, diarrhea, and taste and olfactory dysfunction.

Furthermore, COVID-19 has been reported to cause various dysfunctions of the central nervous system (CNS) [7]. Coronaviruses other than SARS-CoV-2 are known to have neuroinvasive capacities, entering the CNS from the respiratory tract [8]. In addition, it has been reported that patients with severe COVID-19 are more susceptible to CNS disorders than those with non-severe disease [7]. A previous study found that patients, especially those with high-risk factors such as older age and comorbidities (e.g., hypertension) [7], developed not only mild symptoms but also life-threatening complications such as meningitis [9] and acute hemorrhagic necrotizing encephalopathy [10]. Interestingly, in a case report of meningitis, SARS-CoV-2 was not detected in a nasopharyngeal swab but was detected in cerebrospinal fluid [9]. Therefore, it is speculated that damage to the CNS may be caused not only by the effect of cytokine storms as systemic symptoms but also by the direct pathogenic effect of SARS-CoV-2 infection in CNS cells. It is known that appropriate therapeutic intervention for encephalitis is important [11], and factors that easily trigger encephalopathy and encephalitis and those that increase toxicity in the CNS may exist. However, causative evidence and therapeutic strategies for CNS disorders due to SARS-CoV-2 infection remain to be discovered.

## Main text

We focused on CCN1 (cysteine-rich protein 61 (Cyr61)), a member of the matrix secreted protein of the cysteine-rich 61/connective tissue growth factor/nephroblastoma overexpressed (CCN) gene family, which is involved in DNA virus and microbial infection establishment and virulence [12–14]. Furthermore, a recent report showed that Zika virus, an RNA virus, can infect astrocytes in the CNS, which increases CCN1 expression, and that upregulation of CCN1 expression in astrocytes promotes Zika virus replication [15]. This finding suggests that CCN1 is involved not only in the initial period of viral infection but also in increased virulence after the establishment of the viral infection, indicating that CCN1 may play a role in the infection and virulence of viruses, including RNA viruses. SARS-CoV-2 infection may enhance CCN1 expression, as these viruses do. It was recently reported that human small intestine organoids show increased expression of CCN1 at 24 h after SARS-CoV2 infection [16] (Supplementary figure 1).

Although expression of ACE2, the cellular target of SARS-CoV-2 infection, is low in the human brain, there are certain areas where its expression is high ( [17]; Allen Brain Atlas: Human Brain (https://human.bain-map.org/microarray/search)). Interestingly, expression of ACE2 is high in the thalamus [17], which is the region affected by acute hemorrhagic necrotizing encephalopathy [10], and in the choroid plexus, which produces cerebrospinal fluid [17]. In brief, the regions exhibiting high expression of ACE2 and CCN1 in the Allen Brain Atlas are the same as those regions described in case reports [9, 10, 17]. Therefore, the virulence of SARS-CoV-2 may be strong in areas where expression of both ACE2 and CCN1 is high. These findings indicate that CCN1 may play a role as one of the exacerbating factors in COVID-19-associated CNS disorders.

**Fig. 1:**
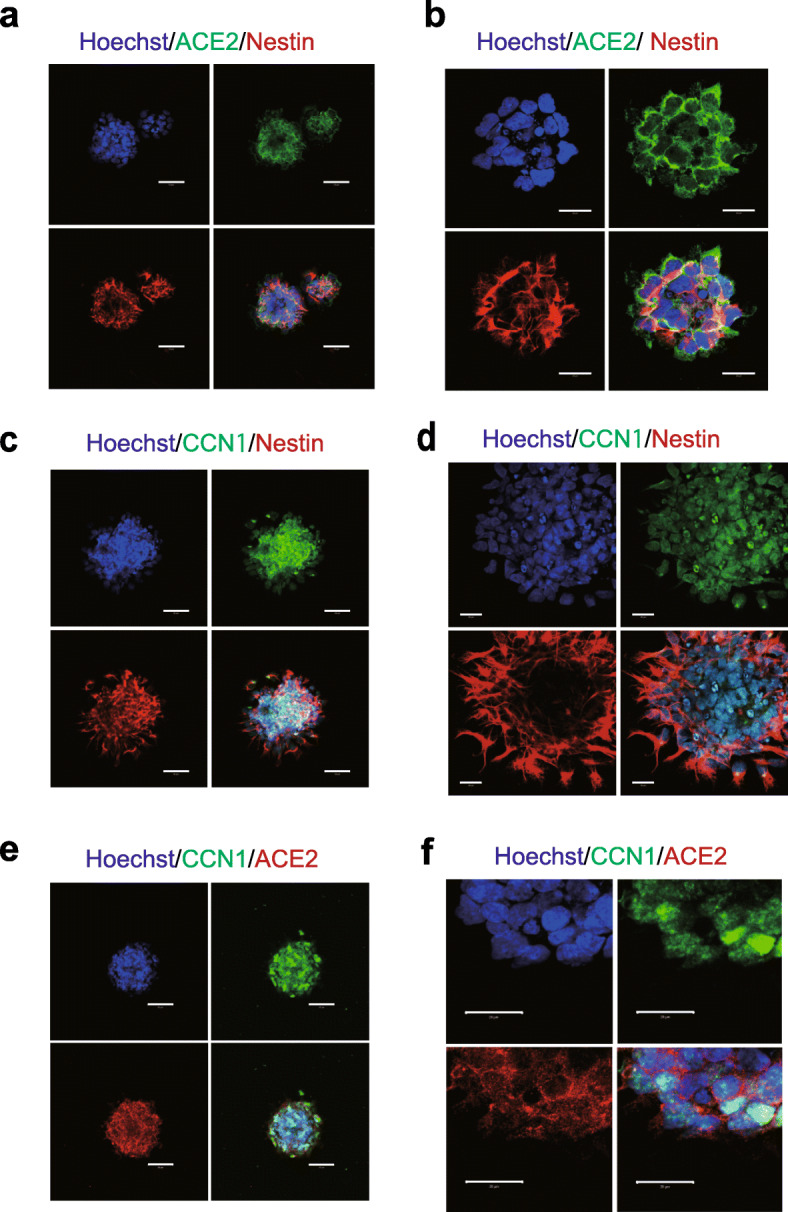
ACE2 and CCN1 are co-expressed in hiPSC-NS/PCs. and **b** Immunostaining images of ACE2 (green) and neural stem/progenitor cell (NS/PC) marker Nestin (red) in hiPSC-NS/PCs. **b** is a high-power field. ACE2 (green) and Nestin (red) are co-expressed. Scale bars represent 50 μm in **a** and 20 μm in **b**. **c** and **d** Immunostaining images of CCN1 (green) and NS/PC marker Nestin (red) in hiPSC-NS/PCs. CCN1 (green) and Nestin (red) are co-expressed. **d** is a high-power field. Scale bars represent 50 μm in **c**, and 20 μm in **d**. **e** and **f** Immunostaining images of CCN1 (green) and ACE2 (red) in hiPSC-NS/PCs. CCN1 (green) and ACE2 (red) are co-expressed. **f** is a high-power field. Scale bars represent 50 μm in **e**, and 20 μm in **f**. Nuclei were counterstained with Hoechst (blue)

As a first step to develop a new model system for COVID-19-associated CNS disorders in vitro and to investigate the possible involvement of CCN1, we investigated expression of ACE2 and CCN1 in neural stem/progenitor cells (NS/PCs) derived from human-induced pluripotent stem cells (hiPSCs) in vitro. We examined expression of ACE2 and CCN1 in NS/PCs differentiated from hiPSCs (hiPSC-NS/PCs; neurospheres) by immunostaining. ACE2 was expressed in hiPSC-NS/PCs, as was CCN1 (Fig. 1a–f). In Fig. 1, we co-stained with Nestin, a neural stem/progenitor cell marker (Fig. 1b, d, and f are high-power fields). ACE2 is stained so as to mostly exclude the nuclei, while CCN1 is localized in both in the cytoplasm and nucleus.

**Fig. 2:**
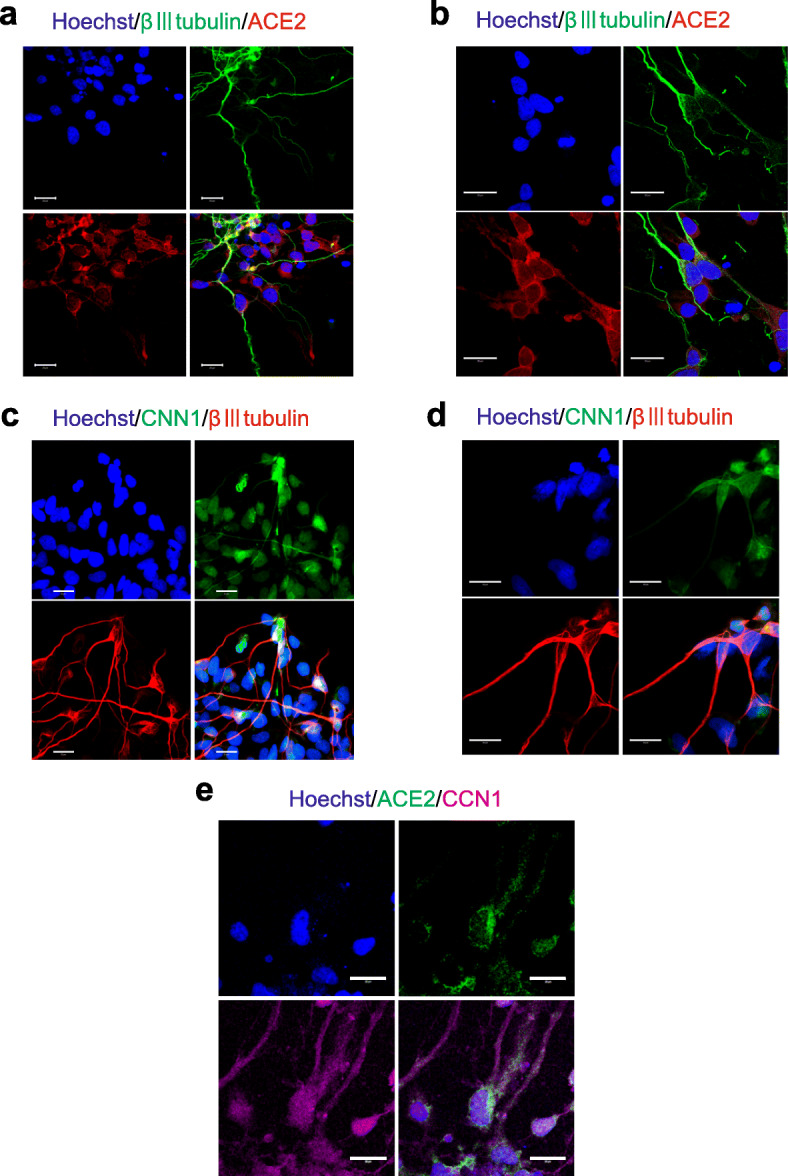
ACE2 and CCN1 are co-expressed in young neurons differentiated from hiPSC-NS/PCs. **a** and **b** Co-immunostaining images with neuronal marker βIII tubulin and ACE2 to confirm that the cells are neurons. The neural marker βIII tubulin (green) and ACE2 (red) in neurons differentiated from hiPSC-NS/PCs. **b** is a high-power field. Scale bars represent 20 μm. **c** and **d** Co-immunostaining images with neuronal marker βIII tubulin and CCN1 to confirm that the cells are neurons. The neural marker CCN1 (green) and βIII tubulin (red) in neurons differentiated from hiPSC-NS/PCs. **d** is a high-power field. Scale bars represent 20 μm. **e** Immunostaining images of ACE2 (green) and CCN1 (magenta) in neurons. ACE2 (green) and CCN1 (magenta) are co-expressed. Scale bars represent 20 μm. Nuclei were counterstained with Hoechst (blue)

Next, neurons differentiated from hiPSC-NS/PCs were immunostained for ACE2 and CCN1, and both were found to be expressed (Fig. 2a–e), co-stained with a neuronal marker, βIII tubulin. Although expression of ACE2 and CCN1 in the adult human brain in vivo varies depending on region, ACE2 and CCN1 were expressed in young neurons derived from hiPSC-NS/PCs. Since the expression of CCN1 and ACE2 is also weakly observed in human ESC-derived neurons in the previous report [18], there may be differences in the level of expression between adult and fetal states. As these neurons or hiPSC-NS/PCs express ACE2, SARS-CoV-2 can infect these cells. In addition, based on reanalysis of the recently published transcriptome database of SARS-CoV-2-infected small intestine organoids [16], the expression level of CCN1 was elevated upon SARS-CoV-2 infection (Supplementary figure 1). Thus, although CCN1 expression is low in neurons without SARS-CoV-2 infection, upregulation of CCN1 may occur after the infection. In addition, it has been suggested that infection with SARS-CoV-2 triggers a cycle of inflammatory reaction via microglia, breakdown of the blood–brain barrier, and further exacerbation of neurological symptoms [19]. Since it is assumed that inflammation caused by microglia is more likely to occur in the elderly, the symptoms may be exacerbated in older patients.

Collectively, hiPSC-NS/PCs expressed a cellular receptor for SARS-CoV-2, ACE2, and could provide an excellent model for COVID-19-associated CNS disorders.

Furthermore, in other series of investigations, we have been conducting experiments on hiPSC-NS/PCs to provide a regenerative treatment for spinal cord injury [20]. Regarding the safety issues of iPSC-based cell therapy, we succeeded in eliminating tumorigenicity and enhancing the therapeutic effect of hiPSC-NS/PCs by pretreating neurospheres with the γ-secretase inhibitor (GSI) DAPT (*N*-[*N*-(3,5-difluorophenacetyl)-*l*-alanyl]-*S* phenylglycine t-butyl ester), which inhibits Notch signaling, before transplantation [21, 22]. To examine the effects of GSI treatment in hiPSC-NS/PCs in more detail, we performed transcriptome analysis after GSI treatment using DAPT and compound 34 [(2*S*,3*R*)-3-(3,4-difluorophenyl)-2-(4-fluorophenyl)-4-hydroxy-*N*-((3*S*)-2-oxo-5-phenyl-2,3-1*H*-benzo[e][1,4]diazepin-3-yl)butyramide]. Compound 34 was also developed as a γ-secretase inhibitor with a benzodiazepine skeleton and was reported in 2003 [23]. It is known to be a higher potent compound than other γ-secretase inhibitors. DAPT is a dipeptide type of GSI and has no benzodiazepine skeleton.

**Fig. 3:**
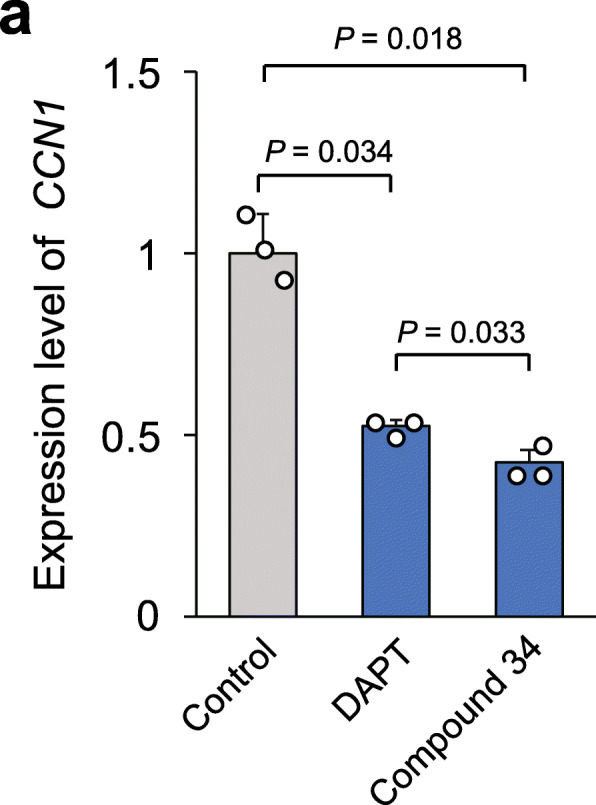
γ-Secretase inhibitor treatments suppress CCN1 expression in hiPSC-NS/PCs. **a** Expression of CCN1 in hiPSC-NS/PCs (neurospheres) is significantly reduced by treatment with a γ-secretase inhibitor (DAPT or compound 34). Compound 34 is more effective than DAPT in lowering CCN1 expression (*n* = 3 independent experiments). As a control sample, dimethyl sulfoxide (DMSO) as a solvent was added instead of GSI. Values in the line graphs represent the mean ± SD.

RNA-seq analyses of single neurospheres were performed after treatment with GSI (DAPT or compound 34), and expression of CCN1 was significantly reduced by approximately half in neurospheres treated with DAPT or compound 34 compared with that in control neurospheres. Moreover, expression of CCN1 was lower when cells were treated with compound 34 than when they were treated with DAPT (Fig. 3a). Currently, the underlying mechanisms of how CCN1 expression is downregulated upon GSI treatment remains to be elucidated. Since compound 34 is synthesized as a higher potent compound [23], compound 34 may be more powerful than DAPT in regulating the expression of these genes.

**Fig. 4:**
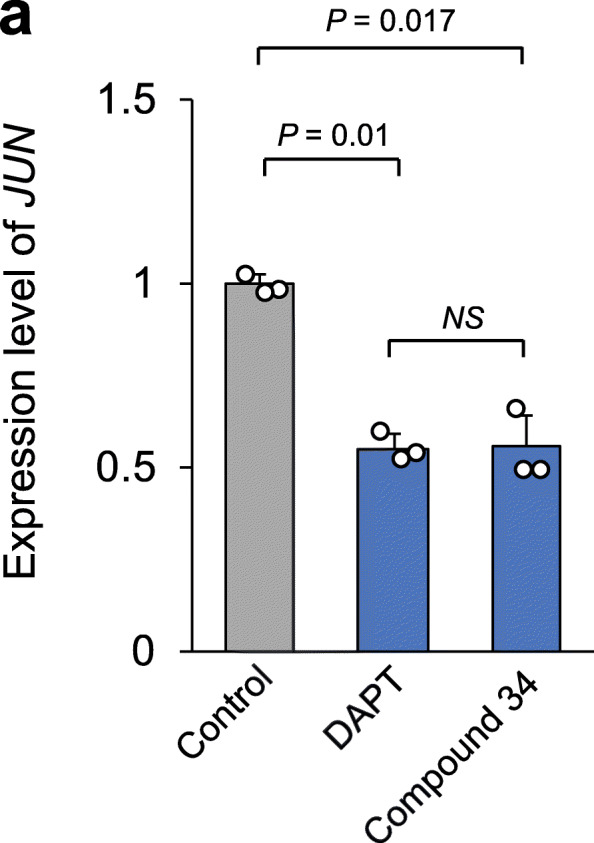
γ-Secretase inhibitor treatments suppress JUN expression in hiPSC-NS/PCs. **a** Expression of JUN in hiPSC-NS/PCs (neurospheres) is significantly reduced by treatment with a γ-secretase inhibitor (DAPT or compound 34) (*n* = 3 independent experiments). As a control sample, dimethyl sulfoxide (DMSO) as a solvent was added instead of GSI. Values in the line graphs represent the mean ± SD.

According to the RNA-seq database for the process of human ESCs differentiation into cortical neurons [18], there is almost no difference in the level of CCN1 expression between undifferentiated and differentiated neurons. Thus, the CCN1 expression level is likely to remain unchanged during the process of differentiation into neurons. Based on this, there is a possibility that CCN1 expression is regulated by a GSI-dependent, but a neuronal differentiation-independent mechanism, thus also different from the regulation of Notch signal which is involved in the neuronal differentiation process. Since it has been reported that suppression of CCN1 expression suppresses replication of RNA and DNA viruses [13, 15], downregulation of CCN1 by GSI may reduce SARS-CoV-2 replication. In addition, a previous report showed that CCN1 enhances virus proliferation and virulence via JNK signaling [13]. Our data confirmed expression of JUN, which is the target substrate of JNK, to be decreased in neurospheres treated with GSI (Fig. 4a). It is also known that enhancement of JNK signaling by CCN1 leads to neuronal cell death [24]. Therefore, GSI (especially, Compound 34) may thus be neuroprotective and have a therapeutic effect on COVID-19-associated CNS disorder by regulating the pathophysiology of SARS-CoV-2 infection and virulence through suppressing the CCN1 expression JNK signaling. Compound 34 is a highly effective GSI that was synthesized as a potential therapeutic agent for Alzheimer’s disease. However, systemic administration of GSI would result in adverse effects by blocking Notch signaling [25]. Thus, it is necessary to investigate the safety, efficacy, and drug delivery of Compound 34 at the preclinical level before actually being applied to treatment of COVID-19-associated CNS disorders.

Furthermore, CCN1 was shown to induce expression and secretion of the inflammatory cytokines IL-1β, TNF-α, and IL-6, and inhibition of CCN1 expression suppressed the actions of these cytokines and attenuated inflammation [26]. Thus, CCN1-induced production/secretion of these inflammatory cytokines might constitute the basis of the enhanced inflammation in various organs, including the lung, gut, and CNS, and cytokine storms [27] associated with COVID-19. In addition, ACE2 itself is known to be involved in inflammatory responses [28]. ACE2 also acts as a cellular receptor of SARS-CoV-2, and increasing CCN1 expression may enhance viral toxicity and induce an inflammatory response. Since ACE2 promotes inflammatory responses by itself [28], it is possible that these factors additively promote the inflammatory response.

The role of SARS-CoV-2 infection-ACE2-CCN1 axis in COVID-19-associated CNS disorders will be unequivocally addressed by in vitro infection experiments for hiPSC-NS/PCs in our future experiments.

## Conclusion

The present study shows that a cellular receptor for SARS-CoV-2, ACE2, is expressed in hiPSC-NS/PCs. Thus, hiPSC-NS/PCs and brain organoids obtained by a long-term culture will provide in vitro experimental systems for SARS-CoV-2 infection in CNS. As mentioned above, CCN1 expression may enhance the viral toxicity of SARS-CoV-2, and its expression may be elevated by SARS-CoV-2. Thus, CCN1 can be expected to be a useful marker and effector for the pathogenetic investigation.

Ideally, the cellular RNA of human neurons infected with SARS-CoV-2 should be analyzed; however, it is difficult to obtain cells from patients with COVID-19. In addition, there are restrictions on SARS-CoV-2 experiments in vivo. Nonetheless, our findings suggest that the damage to the CNS caused by COVID-19 might be reduced by suppressing expression of CCN1, which may thus aid in the development of a therapeutic target for COVID-19-related CNS disorders.

## Methods

### Culture of undifferentiated iPSCs

The human iPSC (hiPSC) line 414C2 [29] was cultured with mitomycin C-treated SNL murine fibroblast feeder cells in standard hESC medium (Reprocell cat. RCHEMD001) containing 0.5% penicillin–streptomycin (Nacalai Tesque) and 4 ng/ml fibroblast growth factor 2 (FGF-2) (PeproTech) in an atmosphere containing 5% CO_2_.

### Formation of neurospheres

hiPSCs (line 414C2) [29] were pretreated for 6 days with 3 μM SB431542 (Tocris) and 150 nM LDN193189 (StemRD). The cells were then dissociated and seeded at a density of 1 × 10^5^ cells per milliliter in ultralow-attachment culture dishes (corning) in a neuronal induction medium consisting of medium hormone mix (MHM) [30] supplemented with 2% B27 supplement without vitamin A (Thermo Fisher), 20 ng/ml FGF, 10 μM Y27632 (Calbiochem), 1 μM retinoic acid (RA; Sigma), 3 μM CHIR (Stemgent) and 10 μM SB431542 (Calbiochem) in a hypoxic and humidified atmosphere (4% O_2_, 5% CO_2_) for 6 days. The spheres formed were passaged by dissociation into single cells and then cultured in the slightly modified neuronal induction medium, MHM supplemented with 2% B27 supplement, 20 ng/ml FGF, Y27632 (Calbiochem), and 1 μM RA (Sigma) for 6 days under 4% O_2_ (hypoxic) conditions.

### Differentiation of neurons derived from hiPSC-NS/PCs (neurospheres)

hiPSC-NS/PCs were plated onto poly-l-ornithine/fibronectin-coated chamber slide glasses (IWAKI, 5732-008). The cells were incubated in medium consisting of medium hormone mix (MHM) [30] supplemented with 2% B27 supplement (Thermo Fisher, 7504-044) and 1% penicillin/streptomycin (Nacalai Tesque, 26253-84) in a humidified atmosphere at 37 °C for 3 days.

### Immunostaining

For immunocytochemistry, samples (neurospheres or neurons) were plated onto poly-l-ornithine/fibronectin-coated chamber slide glasses (IWAKI, 5732-008) and fixed in 4% PFA/PBS for 30 min at room temperature. The slides were rinsed with PBS three times and permeabilized in 0.3% Triton X-100/PBS for 5 min at room temperature. After blocking in TNB buffer for 15 min at room temperature, the slides were incubated at 4 °C overnight with the following antibodies: rabbit polyclonal anti-ACE2 (Abcam, ab15348; 1:250), mouse monoclonal anti-Cyr61 (Santa Cruz, sc-374129; 1:250) or rabbit monoclonal anti-Cyr61 (Cell Signaling Technology, 14479S; 1:250), and mouse anti-Nestin (MERCK, MAB5326; 1:500) or rabbit anti-human Nestin (IBL, N1602; 1:500), mouse monoclonal anti-β-tubulin III (tuj1) (Sigma-Aldrich, T8660; 1:500). After washing three times with PBS, the samples were incubated for 60 min at room temperature with secondary antibodies conjugated with Alexa 488 (Thermo Fisher Scientific, A-11034 or A-11001) or Alexa 555 (Thermo Fisher Scientific, A-21429 or A-21434), followed by nuclear counterstaining with Hoechst 33258 (Sigma-Aldrich, B2883; 10 μg/ml). The samples were analyzed with a confocal laser scanning microscope LSM700 (Carl Zeiss).

### Treatment of hiPSC-NS/PCs (neurospheres) with γ-secretase inhibitors (GSIs)

The small-molecule GSIs *N*-[*N*-(3,5-difluorophenacetyl)-*l*-alanyl]-*S*-phenylglycine t-butyl ester (DAPT; Sigma-Aldrich) and (2*S*,3*R*)-3-(3,4-difluorophenyl)-2-(4-fluorophenyl)-4-hydroxy-*N*-((3S)-2-oxo-5-phenyl-2,3-1*H*-benzo[e][1,4]diazepin-3-yl)butyramide (compound 34: Santa Cruz) were dissolved in DMSO. DAPT was added to neurospheres at a final concentration of 10 μM. Compound 34 was added to neurospheres at a final concentration of 2 μM. The neurospheres were cultured for 24 h.

### RNA sequencing of neurospheres

Neurosphere sequencing was performed by Takara Bio Inc. (Kusatsu, Japan). Purified RNA was resuspended in Clontech buffers for mRNA amplification using 5′ template switching PCR with a Clontech SMART-Seq v4 Ultra Low Input RNA Kit according to the manufacturer’s instructions. Amplified cDNA was fragmented and linked with dual-indexed barcodes using Illumina Nextera XT DNA Library Prep Kits. The libraries were validated using an Agilent 4200 TapeStation, pooled and sequenced with an Illumina NovaSeq 6000. Gene expression was drawn on the TPM value.

### Statistics

Statistical significance was determined by one-way ANOVA and Dunnett’s T3 post hoc test. Throughout the study, *P* values < 0.05 were considered statistically significant. Values in bars represent the mean ± SD in figures.

## Supplementary information

**Additional file 1: Figure S1.** Expression of CNN1 (Cyr61) increased 24 hours after SARS-CoV-2 infection of small intestinal organoids. These data were obtained from reference [16]. Values in the line graphs represent the mean ± SD.

## Abbreviations

ACE2: Angiotensin-converting enzyme 2
CCN1 (Cyr61): CCN family member 1 (cysteine-rich protein 61)
CNS: Central nervous system
COVID-19: Coronavirus disease 2019
DMSO: Dimethyl sulfoxide
GSI: γ-Secretase inhibitor
hiPSCs: Human-induced pluripotent stem cells
NS/PCs: Neural stem/progenitor cells
hiPSC-NS/PCs: NS/PCs differentiated from hiPSCs
SARS-CoV-2: Severe acute respiratory syndrome coronavirus 2
