# Supplementary material for: Expression of ACE2 and a viral virulence-regulating factor CCN family member 1 in human iPSC-derived neural cells: implications for COVID-19-related CNS disorders
Source: Inflamm Regen. 2020 Sep 11;40:32. doi: 10.1186/s41232-020-00143-6 (PMC7485212; doi:10.1186/s41232-020-00143-6)
Supplement: Supplementary file 1 [file 41232_2020_143_MOESM1_ESM.docx]

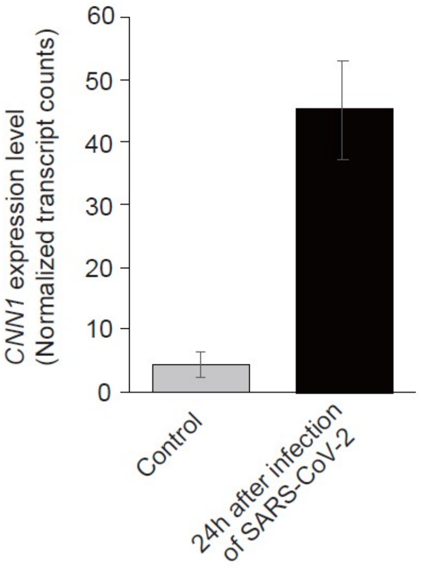

(This graph was obtained by the re-analysis of transcrioptome data described in
"Lammer MM et al. SARS-CoV-2 productively infects human gut enterocytes." Science, 2020.)
